# Supplementary material for: Estimating protein complex model accuracy using graph transformers and pairwise similarity graphs
Source: Bioinform Adv. 2025 Jul 29;5(1):vbaf180. doi: 10.1093/bioadv/vbaf180 (PMC12342149; doi:10.1093/bioadv/vbaf180)
Supplement: vbaf180_Supplementary_Data [file vbaf180_supplementary_data.pdf]

Supplementary Material

Jian Liu, Pawan Neupane, Jianlin Cheng

July 23, 2025

Algorithm S1 Pairwise Similarity Graph Construction and Subgraph Sampling

Input: Pairwise similarity matrix M for all decoy pairs; number of subgraphs N
Construct pairwise similarity graph $G = (V, E)$, where each node $v \in V$ is a decoy, and an edge $(v_i, v_j) \in E$ exists if $M(v_i, v_j) > 0.5$
Compute the overall average similarity score: $\mu \leftarrow \text{mean}(M)$
Initialize subgraph set: $\mathcal{G} \leftarrow \emptyset$
if $\mu < 0.8$ **then**
  Determine optimal number of clusters k by maximizing the silhouette score for $k \in \{2, \ldots, 9\}$ using KMeans on M
  Apply KMeans clustering with k clusters to group structurally similar decoys
  Let $s \leftarrow \lfloor 50/k \rfloor$ denote the number of samples per cluster
  for $i = 1$ to N **do**
    Initialize subgraph node set $V_S \leftarrow \emptyset$
    for $j = 1$ to k **do**
      Randomly sample up to s decoys from cluster j and add them to V_S
    end for
    Define subgraph $G_S^{(i)} = (V_S, E_S)$ where E_S includes edges from E between nodes in V_S
    Add $G_S^{(i)}$ to $\mathcal{G}$
  end for
else
  for $i = 1$ to N **do**
    Randomly sample up to 50 decoys to form node set V_S
    Define subgraph $G_S^{(i)} = (V_S, E_S)$ where E_S includes edges from E between nodes in V_S
    Add $G_S^{(i)}$ to $\mathcal{G}$
  end for
end if
Output: Set of N sampled subgraphs $\mathcal{G} = \{G_S^{(1)}, G_S^{(2)}, \ldots, G_S^{(N)}\}$ from G

Table S1: Hyperparameter search space explored during model fine-tuning. Abbreviations used in subsequent tables are shown in parentheses.

**Hyperparameter (abbr.)**	**Candidate Values**
Number of attention heads (`heads`)	4, 8
Number of graph transformer layers (`layers`)	2, 3, 4, 5
Dropout rate (`dp`)	0.1, 0.2, 0.3, 0.4, 0.5
MLP dropout rate (`mlp_dp`)	0.1, 0.2, 0.3, 0.4, 0.5
Hidden dimension (`hid_dim`)	16, 32, 64
Weight of the pairwise loss (w_{pair})	`auto`, 0.01, 0.05, 0.1, 0.2, 0.3, 0.4, 0.5, 0.6, 0.7, 0.8, 0.9, 1
Optimizer (`opt`)	`AdamW`, `SGD`
Learning rate (`lr`)	1e-5, 5e-5, 1e-4, 5e-4, 1e-3
Weight decay (`wd`)	0.01, 0.05
Layer normalization (`LN`)	False, True
Batch size (`batch`)	256, 400, 512

Table S2: Selected hyperparameter values and model size (in trainable parameters) for each fold of GATE-Basic. Abbreviations are defined in Table S1.

Fold	heads	layers	dp	mlp_dp	hid_dim	w_{pair}	opt	lr	wd	LN	batch	agg	Params
fold0	8	2	0.3	0.1	64	0.7	AdamW	0.001	0.01	True	400	median	122K
fold1	4	2	0.3	0.5	16	0.5	AdamW	0.0001	0.01	False	400	median	9K
fold2	4	5	0.3	0.2	32	0.01	AdamW	0.0001	0.05	False	400	median	78K
fold3	4	2	0.3	0.5	16	0.01	AdamW	0.001	0.01	True	400	mean	8K
fold4	8	3	0.1	0.2	64	0.7	SGD	0.0001	0.01	False	400	median	182K
fold5	8	5	0.5	0.5	32	1	AdamW	1e-05	0.01	False	400	median	78K
fold6	4	2	0.3	0.4	16	1	SGD	0.001	0.01	False	400	mean	9K
fold7	4	2	0.1	0.1	64	0.05	AdamW	5e-05	0.05	True	512	mean	122K
fold8	8	2	0.1	0.3	64	0.2	AdamW	5e-05	0.05	True	512	mean	122K
fold9	8	2	0.5	0.4	64	0.2	SGD	0.0001	0.01	False	400	mean	123K

Table S3: Selected hyperparameter values and model size (in trainable parameters) for each fold of GATE-GCP. Abbreviations are defined in Table S1.

Fold	heads	layers	dp	mlp_dp	hid_dim	w_{pair}	opt	lr	wd	LN	batch	agg	Params
fold0	8	2	0.5	0.1	64	1	AdamW	0.001	0.01	True	512	mean	122K
fold1	8	5	0.1	0.2	64	0.9	SGD	5e-05	0.05	True	400	median	298K
fold2	4	4	0.5	0.2	32	1	AdamW	0.0001	0.01	True	256	median	62K
fold3	4	5	0.1	0.4	16	0.1	AdamW	0.0005	0.01	True	512	median	20K
fold4	4	5	0.2	0.1	64	1	SGD	5e-05	0.01	True	256	mean	298K
fold5	4	3	0.5	0.5	32	0.9	AdamW	1e-05	0.05	False	400	median	47K
fold6	4	5	0.1	0.1	16	1	SGD	5e-05	0.05	False	256	mean	21K
fold7	4	4	0.2	0.1	64	0.7	AdamW	0.0001	0.01	False	512	mean	241K
fold8	4	2	0.3	0.1	64	0.8	AdamW	5e-05	0.05	True	512	median	122K
fold9	8	2	0.5	0.4	64	0.5	SGD	0.0001	0.01	False	400	mean	123K

Table S4: Selected hyperparameter values and model size (in trainable parameters) for each fold of GATE-Advanced. Abbreviations are defined in Table S1.

Fold	heads	layers	dp	mlp_dp	hid_dim	w_{pair}	opt	lr	wd	LN	batch	agg	Params
fold0	4	5	0.2	0.2	64	0.3	AdamW	1e-05	0.05	False	400	median	301K
fold1	4	5	0.1	0.4	64	0.4	SGD	1e-04	0.01	False	256	mean	301K
fold2	4	5	0.5	0.2	64	0.5	AdamW	5e-05	0.01	True	256	median	298K
fold3	4	2	0.3	0.5	16	0.5	AdamW	1e-04	0.01	False	400	mean	9K
fold4	4	5	0.2	0.1	64	0.4	SGD	5e-04	0.01	False	400	median	301K
fold5	8	5	0.4	0.5	64	auto	AdamW	1e-05	0.01	False	400	mean	301K
fold6	4	2	0.3	0.4	16	1	SGD	1e-03	0.01	False	400	mean	9K
fold7	4	4	0.1	0.2	64	0.5	AdamW	1e-05	0.05	True	512	median	240K
fold8	4	4	0.1	0.2	16	0.05	AdamW	5e-04	0.01	False	512	mean	17K
fold9	4	5	0.5	0.2	64	0.9	SGD	1e-05	0.01	False	256	mean	301K

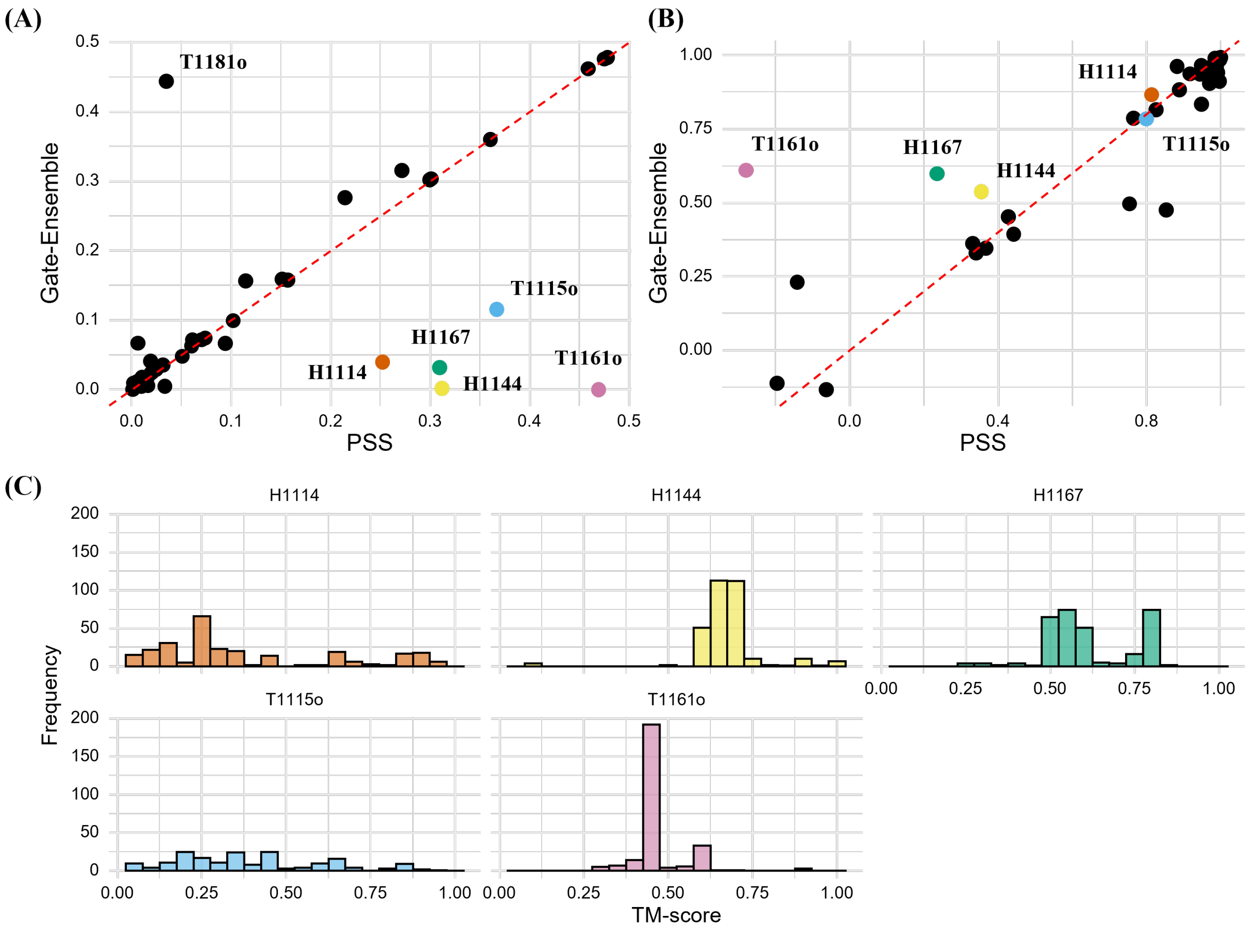

Figure S1: Per-target performance comparison between the PSS and GATE-Ensemble on CASP15 complex targets. (A) Ranking loss of GATE-Ensemble plotted against that of PSS; (B) Pearson's correlation of GATE-Ensemble plotted against that of PSS; (C) the histogram (distribution) of true TM-scores of the decoys for H1114, H1144, H1167, T1115o, T1161o.

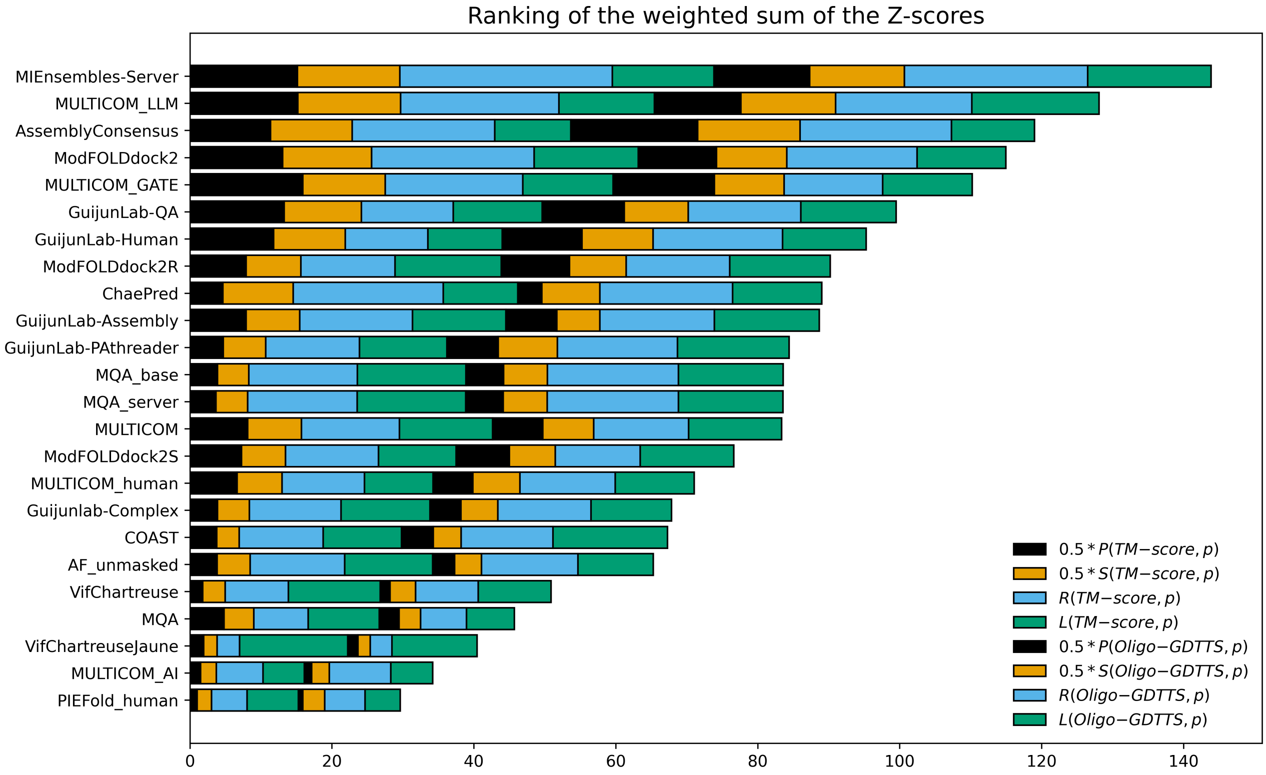

Figure S2: The overall performance of 23 CASP16 predictors in estimating the accuracy of the structural models of 36 out of 38 CASP16 multimer targets according to the z-scores of multiple evaluation metrics (i.e., Pearson's correlation, Spearman's correlation, AUC, and ranking loss) in terms of both TM-score and oligomer GDT-TS score. Each kind of z-score is denoted by a colored bar. The predictors are ordered according to the weighted sum of all the z-scores. The publicly available code from the CASP16 EMA GitLab repository to perform our own analysis and generate the figure: `https://git.scicore.unibas.ch/schwede/casp16_ema`.

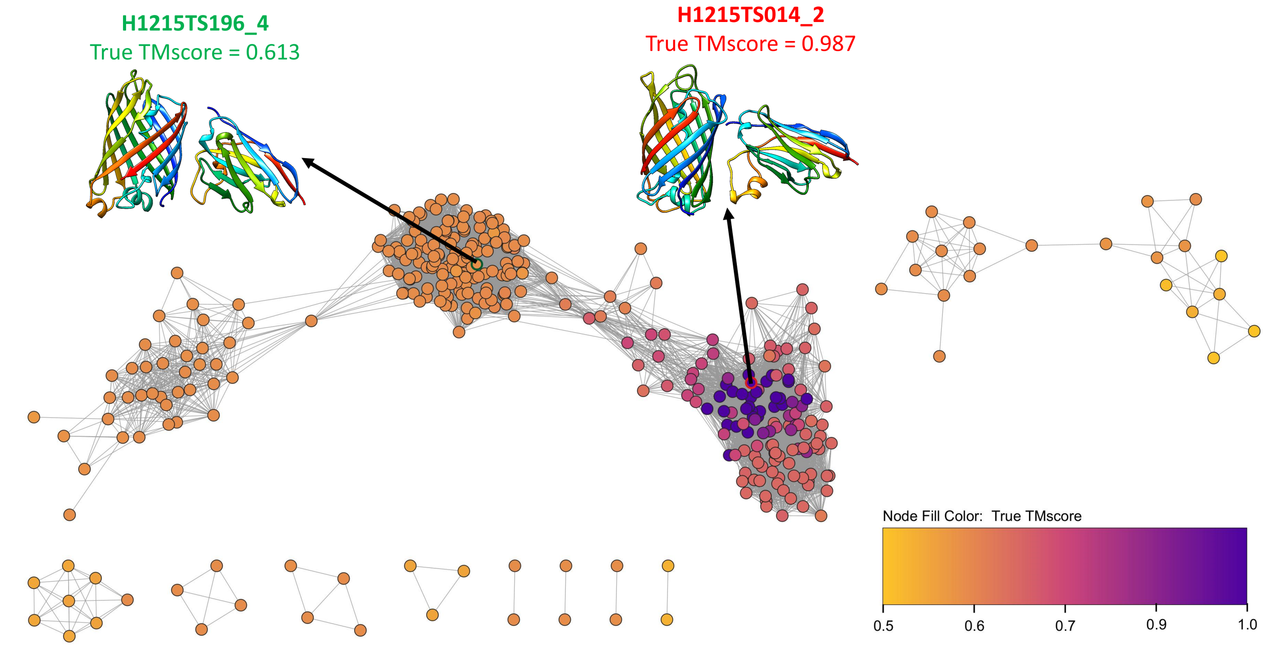

Figure S3: The pairwise similarity graph for the structural modes (decoys) of CASP16 target H1215. The top 1 decoy selected by the average pairwise similarity score (H1215TS196_4, true TM-score = 0.613) and MULTICOM_GATE (H1215TS014_2, true TM-score = 0.987).
